# Perceptions of HIV self-testing promotion in black barbershop businesses: implications for equitable engagement of black-owned small businesses for public health programs

**DOI:** 10.1186/s12889-024-19973-x

**Published:** 2024-09-09

**Authors:** Donaldson F. Conserve, Allison Mathews, Samuel Janson, Ucheoma Nwaozuru, Larissa Jennings Mayo-Wilson, Tiarney D. Ritchwood, Aima A. Ahonkhai, Hassim Diallo, Tayo Korede, Arona Dieng, Angela F. Randolph, BRIDGE Research Team

**Affiliations:** 1 Milken Institute School of Public Health, The George Washington University, Washington, DC USA; 2 University of North Carolina Gillings School of Global Public Health, Chapel Hill, NC USA; 3 Community Expert Solutions, Inc, Winston-Salem, NC USA; 4 Massachusetts General Hospital, Boston, MA USA; 5 School of Public Health, University of Maryland, College Park, MD USA; 6 University of Central Lancashire, Preston, Lancashire, UK; 7 Babson College, Babson Park, MA USA; 8 Building Research and Implementation to Drive Growth and Equity (BRIDGE) Research Lab, Milken Institute School of Public Health, Washington, DC USA

**Keywords:** HIV self-testing, Men’s health, HIV, Business venues, Implementation science, Entrepreneurship

## Abstract

**Background:**

HIV self-testing (HIVST) offers an innovative and promising approach to increasing HIV testing among Black men in the United States, a population disproportionately affected by HIV. However, engaging Black men in traditional HIV prevention programs has been challenging due to stigma, medical mistrust, and limited access to preventive health services. This formative qualitative study aimed to explore the potential of utilizing barbershops as an example of a nontraditional healthcare venue to promote and distribute HIVST.

**Methods:**

Four virtual focus group discussions (FGDs) consisting of 19 participants in North Carolina were conducted with Black men, including barbershop business owners, barbers, and their customers, to assess perceptions of HIVST and the acceptability of partnering with barbershop businesses to promote HIVST. FGDs were digitally recorded, transcribed, and analyzed using a deductive coding approach to thematic analysis.

**Results:**

Participants reported that the trusting relationship between barbers and their customers, which may not exist between Black men and health care providers, is a facilitator of collaborating with barbershop businesses to reach Black men for HIVST distribution. Participants recommended providing education for barbers on the use of HIVST, as well as how to inform self-testers about linkage to care following HIVST to build the credibility of the barbers in delivering the intervention. Participants also raised the issue of the cost of HIVST to barbershop customers as a potential barrier to implementation, as well as the possibility that the implementation of such interventions could be seen as out of place in a barbershop business venue. Participants also expressed a strong belief that compensation to barbershops and their employees should accompany any intervention.

**Conclusion:**

These findings suggest that barbershop business venues may provide an appropriate venue for HIVST promotion and distribution, though factors like cost, training, and incentivization of implementers are necessary to consider in implementation planning. Furthermore, partnerships between public health actors and the business community must be built on equitable engagement to ensure the long-term viability of these critical initiatives.

## Background

The disproportionate number of Black men diagnosed with HIV in the United States is undeniable. Black Americans account for just 13% of the population, but 40% of new HIV cases [1]. Among those new cases, three-quarters are Black males, the majority of whom are men who have sex with men (MSM) [1]. The unequal share of the HIV burden experienced by Black men is driven in part by the social determinants of health [2]. Black Americans experience higher levels of poverty (22%), unemployment (11%), and food insecurity (24%) than the national averages [3], and inhibit access to preventive HIV care and treatment for Black men [2, 4, 5]. In addition to structural barriers, social challenges such as high levels of internalized stigma have adverse effects on HIV outcomes for Black men, and healthcare providers often find the initiation of sexual behavior-related conversations with Black men to be a challenge [6, 7]. This convergence of factors has contributed to the low uptake of HIV prevention methods, including pre-exposure prophylaxis medication (PrEP). Among Black Americans at high risk for HIV, only 8% have been prescribed PrEP, compared to 23% across races [8].

Despite the elevated risk for seroconversion among Black men, their rates of HIV testing in the United States are low, and research regarding Black men and HIV testing has identified an insufficient volume of interventions tailored to their needs [9]. Nearly one-third (31.2%) of Black men (aged 15–44) report never having been tested for HIV, and rates of never-testing are even lower among young Black men (aged 15–24) at 66.1% [10]. Black men generally access preventive health care at low rates, and while most HIV testing takes place in clinical settings, these venues pose access barriers to this population due to cost and lack of insurance [11]. Additional individual-level barriers to HIV testing among Black men include internalized stigma, low perceived risk of HIV, and fear of testing [11–14]. Given this combination of risk and unique barriers, tailored HIV prevention interventions are needed to curb the HIV epidemic in Black men [15].

One innovative strategy to promote HIV testing among Black men is oral HIV self-testing (HIVST) kits, which have emerged as a solution to low testing uptake among hard-to-reach populations [16]. Scholars agree that HIVST can increase the awareness of people with positive statuses if the tests are affordable or fully subsidized for people at high risk for acquiring HIV [17]. Black men in the United States have generally expressed acceptability of HIVST, preferring privacy and convenience compared to facility-based HIV testing [18–20]. However, interventions to promote awareness and uptake of HIVST among Black American men have been limited. The few interventions piloted in the United States have utilized peer-led and digital health strategies but have not yet evaluated the possibility of barbershops and other untraditional Black business venues trusted by community members for HIVST education and promotion [18, 20]. Furthermore, these previously piloted HIVST promotion interventions have focused on Black MSM, whereas barbershops service a broader range of the Black male population.

### Black Barbershop Businesses and Health Equity

Among all Black-owned businesses in the United States that contribute to the cultural and economic vitality of the nation, the Black barbershop businesses stand alone. The history of Black barbering is deeply entwined with the painful racial history of the United States, as historical records show that enslaved Black men were often taught barbering as a way of generating income for their owners [21]. However, barbering also soon became an avenue of Black liberation, as enslaved men used their skills cutting hair to earn sufficient income to purchase their freedom [22]. Following emancipation, Black barbershops began to proliferate and grew alongside the Black middle class, which eventually began to diversify the barbershops’ clientele away from solely White men [22]. This growth led to substantial wealth acquisition for the Black community, and some of the first Black millionaires in the 20th century included Alonzo Herndon and Willie Morrow, who both gained a substantial portion of their wealth in the barbering industry [23–25]. The Black barbershop also occupies a significant social space in the Black community of the United States and played a prominent role in the Civil Rights Movement of the 1960s, and more recently the COVID-19 pandemic response [21, 26–29].

In addition to expanding employment opportunities to Black barbers and their related economic gains for the Black community, Black barbershop businesses have also served as vital sources of health information and have served as venues for health promotion interventions including cardiovascular disease, diabetes, and cancer screenings, as well as to initiate conversations on mental health [28, 30–37]. Rather than relying on formal healthcare providers, who face high levels of mistrust in the Black community [32, 38], Black-owned barbershop businesses and their employees have participated in leading health promotion interventions, including disease screenings and health education [39–44]. These interventions are also bolstered by the increasing use of social media among Barbershop business owners to enhance the reach of their business and promote their services [45]. One study promoting physical activity in Black barbershops found an increase in physical activity and decreased risk factors for many common chronic diseases among clients of those barbershops [46]. Another more recent example is the active role of barbershop businesses in increasing awareness of the COVID-19 vaccine and, in some cases, providing a venue for patrons to receive their shots [29]. These efforts were spearheaded by a White House initiative in collaboration with the National Association of County and City Health Officials in response to lagging rates of COVID-19 vaccination in the Black community [47]. Black-owned barbershop businesses are successful health intervention venues due to the high cultural significance they hold for Black men, the positive and trusting relationship between Black men and their barbers, and their historical reputation as sources of health information [26, 33, 43, 48].

### Challenges in Black Entrepreneurship and implications for Partnership

While there have been myriad examples of effective collaboration between Black barbershops and public health institutions, ongoing partnerships are not guaranteed, given the precarity of many Black-owned businesses in the United States. Black entrepreneurs start with less financial capital and have less access to financial capital from lending institutions and equity investors than their white peers [49]. Black entrepreneurs are also more likely than their white peers to have their expertise doubted when trying to grow their business by competing for contracts with larger firms [50]. Black-owned businesses are more vulnerable to external economic shocks, and are at greater risk of closing their doors during periods of economic hardship, such as the COVID-19 pandemic [51]. Due to the obstacles Black entrepreneurs face, Black-owned businesses are more likely to fail [52]. Factors that affect careers and entrepreneurial success like educational background and economic inequality also affect social concerns like health, economic growth, and crime [53].

However, there has been minimal consideration of these challenges as part of the evaluation of the success and sustainability of barbershop-based health interventions. A recent systematic review of 14 Black barbershop- and salon-based interventions found that 3 of the studies included in the review did not report on compensation for either barbershop staff or their customers, 4 reported compensation only to barbershop customers receiving the intervention, and 7 reported either compensation to either barbershop staff or both staff and their customers [36]. Another recent scoping review provided an overview of the implementation of 13 barbershop-based health promotion interventions for Black men using the RE-AIM framework [37]. This review gave important recommendations for the future reporting of results from such interventions such as their impact on mental health outcomes and a more robust reporting on implementation strategies for shared learning [37]. However, the authors did not call for the inclusion of reporting compensation, or other strategies for evaluating the equitable engagement of the participating business, despite these factors being critical to the adoption of future interventions and their ongoing maintenance. These challenges are part of the landscape in which partnerships between public health agents and Black-owned barbershops operate, and thus must be considered in evaluating the feasibility of health promotion interventions.

### Study objectives

This qualitative study aimed to explore the acceptability and feasibility of leveraging Black owned barbershop businesses within Black communities to promote HIVST and provide relevant information about HIV to Black men. This work was informed by a prior study [54] for which crowdsourcing contests were used to elicit community-based ideas on how to promote HIVST kits in Black communities in the Triangle area of North Carolina, including Raleigh, Durham, and Chapel Hill. The top ideas from the crowdsourcing contests recommended using information booths located at community locations, such as the library, near basketball parks, and barbershop businesses to promote HIVST uptake and general HIV knowledge [54].

## Methods

### Study setting and participants

To recruit participants, we used flyers, emails, social media, phone calls, and in-person visits to Black-owned barbershops in the Triangle area, including those who had previously participated in a heart health intervention [55]. Flyers were placed at barbershops and in local restaurants and distributed among the members of a local running group. Social media posts were also made on Instagram. Focus groups were open to Black (African, African American, and Caribbean American) men aged 18 and above who lived in North Carolina’s Triangle area. Additional participants were recruited from a convenience sample of Black men’s running groups and social networks. Participants did not have to disclose their HIV status. The University of North Carolina at Chapel Hill’s Institutional Review Board gave their approval to the study. This research was carried out in North Carolina’s Triangle region, which includes Durham, the state’s sixth most HIV-positive county (24.3 per 100,000 persons) [55]. Blacks/African Americans account for 62.3% of all cases in the Triangle region, while making up just 31% of the population [56, 57]. In a study of Black young adults in Durham, North Carolina, it was shown that Black men had lower HIV testing rates than Black women because they were less inclined to participate in regular screenings in clinical settings [58].

### Data Collection

Four virtual focus group discussions (FGDs) consisting of 19 participants total were held using Zoom video conferencing from January through June 2019 [59, 60]. The participants included including barbershop business owners, barbers, and barbershop customers. Each conversation was audio-recorded and lasted between 1.5 and 2 h. To ensure confidentiality, FGD participants were offered the option of participating without video and had their names changed to their allocated number on their login screens. Each participant provided verbal informed consent. The discussion was moderated by two PhD-level researchers (DFC and AM) with extensive qualitative research experience. The moderators demonstrated an HIVST kit and stated that researchers were attempting to figure out if setting up HIVST information booths in places like barbershops would be an appropriate way to encourage HIV testing in their community. The FGD guide was adapted from a study that developed an HIVST education and promotion project [11]. Items to explore HIV self-testing perceptions included, “*How many of you would want to use an HIV self-test kit*? *Why would you want to use an HIV self-test kit*?” Items included to assess perceptions of barbershop-based HIV self-testing education and promotion were included: “*What are some potential concerns and challenges you think men may have with receiving HIV self-testing information in barbershops*?”

### Data Analysis

The audio recordings of the FGDs were anonymously transcribed verbatim. Two study team members analyzed the data independently using both codes developed a priori from a previous study [11], followed by an deductive coding process. The codes developed a priori and included the following: General perceptions of HIVST, positive and negative perceptions of specific HIVST promotion, and finally, recommendations on how to promote HIVST in barbershops. Relevant themes and responses were identified, grouped, and compared. We also conducted an deductive thematic analysis, where the codebook was expanded throughout the coding process to match emerging themes [61]. This process included reading the transcribed data line by line for familiarization, generating the initial codes, and identifying and reviewing themes. The deductive coding was guided by the Consolidated Framework for Implementation Research (CFIR) 2.0 [62]. CFIR 2.0 is useful for eliciting information regarding individual, institutional, and societal factors that can influence intervention or policy implementation across different contexts [62]. CFIR 2.0 was also selected due to its categorization of implementation-level factors by various domains of implementation, such as inner setting, outer setting, characteristics of the intervention, etc. The responses to the open-ended questions within the FGD were analyzed using qualitative thematic analysis. Discrepancies between coders were identified, and a third member of the study team made the final decision on how the response should be coded.

## Results

### General perceptions of HIVST

We first gathered viewpoints on the benefits and drawbacks of introducing HIVST education and promotion into barbershops as a secondary prevention measure, whereby diseases are detected, in contrast to primary prevention, which seeks to prevent the transmission/development of a disease. In the case of HIV, primary prevention may include the use of condoms or PrEP. For advantages, participants cited the close contact between barber and client, rather than between doctor and patient. Participants expressed a feeling that the barbershop is a discreet place to get self-testing kits and HIV education, given that it is not traditionally associated with HIV care. Participants noted potential drawbacks of the barbershop as an HIVST education and distribution point, including the additional cost of an HIVST kit and the possibility that barbers may not have sufficient scientific knowledge to teach customers about HIVST.

### Facilitators of barbershop businesses as venues for HIVST promotion

Participants cited several perceived facilitators of barbershop business-based HIVST promotion and why such marketing and distribution are generally acceptable in community venues. Two key themes emerged: (a) the trusting relationships customers have with barbers, and (b) barbershop businesses as decentralized and non-stigmatized locations for providing HIV prevention information.

### Trusting relationship with barbers

Participants stated that the trusting relationship between customers and their barber makes it more acceptable for them to receive HIVST information from them because they feel a greater sense of equality when compared to a healthcare provider:

It’s a relationship where people feel more on equal footing, you know, more so than ‘I’m a doctor’ this expert just kind of rushing through things.

Other participants agreed and expanded on how clients’ trusting relationship with their barber increased acceptance of discussing HIVST in this setting, with one participant saying:

Most people tend to have a very specific relationship with their barber … so the fact that they are already trusting them with their style usually opens up for a very strong relationship … it would definitely be more successful and more impactful because you have somebody you already know and that makes a huge difference compared to someone you don’t know.

### Barbershop businesses as decentralized and non-stigmatized locations for providing HIV prevention information

A few participants agreed that, when compared to a pharmacy or a doctor’s office, barbershop businesses would be a much more appropriate venue to promote HIVST and sexual safety guidelines as the barbershop setting can foster a more informal, voluntary, and stigma-free discourse about HIVST:

At the barbershop you don’t have to worry about if someone else sees it or anything because it is already in the barbershop. It is something that can start up a conversation in the barbershop. So, it’s a little less stigmatized there just because you can start up that conversation better than if you’re in the pharmacy….

Some of the participants perceived barbers would be amenable to promoting HIV testing at their barbershops and that they would even be interested in acquiring HIV testing kits to offer as an addition to the condoms and HIV testing information that some barbershops currently have:

I think it would definitely be okay to have them at the barbershop. The barbershop is going to buy them, and then sell them again inside the barbershop. I usually go to the barbershop [that] has free condoms and they have the little pamphlets about getting HIV testing so why not take that extra step and have them there, you know?

### Potential barriers to barbershop business-based HIVST promotion

The potential barriers to the use of barbershops for HIVST promotion included: perceived cost constraints, concerns about barbers’ knowledge of HIV, and whether the HIVST kit may lose impact in the barbershop setting. Some participants thought that HIV testing in barbershop businesses could be beneficial, but that there were certain conditions that would need to be met for it to be successful in the community. These conditions included giving out the HIVST kits for free or as part of a package deal with a haircut and incentivizing and educating barbers to advocate and promote HIV testing.

### Cost of HIVST + haircut

Most participants agreed that if tests were not free, people would be discouraged when coming to barbershop businesses and would not want to spend extra money on top of the cost of their haircut. Participants suggested that to potentially combat the cost problem, a discount could be offered on top of the haircut or the haircut and HIVST kit could be bundled into one price.

I didn’t come here to buy it [HIVST], so a way to promote it could be wrapped up in the cost of the haircut you know maybe if you buy the test, you get a free haircut or you could get a discounted haircut or something like that… That way it’s more appealing.

### Barbers will not have scientific knowledge to support claims

Participants also provided feedback related to the potential for concerns about the validity of the information provided by barbers and the credibility of barbers for providing this type of information. One participant noted the lack of formal education for barbers in delivering health education may lead to skepticism among clients:

I would definitely ask for fact checking. Let’s say there was somebody who was credentialed in some kind of medical field or health services or something like that and let’s say there’s another guy in the barber shop and he’s spitting information. I’d be like, ‘where’d you learn that? Oh, I went to school for it.’ At that point, I’m going to be less likely to question it than being someone who’s read it themselves… So if a brother was in (the barbershop) there telling me, you should use this test instead of (another test)… I’m going to say well what gives you the right to provide a medical opinion?

### HIVST could lose impact in barbershop business venues

One participant voiced concern that since barbershops frequently advertise other products and information, people might habituate to the availability of HIVST kits, such that the kits would be viewed as one of many products sold at barbershops and thus may not be as highly valued within the community:

You know it’s a bad thing; I think it will lose its integrity in terms of this initiative because once it becomes just another thing to sell in the barbershop in terms of ‘I got some bootleg videotapes and I got these HIV testing kits too’, you know, ‘and these socks I just got from target’ you know to sell you five packs for a dollar you know kind of thing. So I think you just have to be just sensitive about that.

Another participant agreed, and mentioned that the inclusion of HIVST promotion in barbershop businesses could work against the professional environment the barber is trying to establish:

The public and the barbering industry and the barbers is already fragile enough in terms of trying to maintain a professional environment and business, and I think we’re not even, myself, doing a good enough job advising people in healthcare and practices as a true professional in that specific field. I think that adding this in is another way to you know add on to the “hustle” of barbering.

### Recommendations on how to promote HIVST in barbershop businesses

Participants provided several recommendations for considerations in implementing a barbershop business-based strategies for HIVST: (a) provision of monetary incentives for barbershop businesses involvement, (b) factoring in stigma surrounding HIV and HIVST, (c) counseling for post-positive test results and linkage to follow-up care.

### Monetary incentive for barbershop businesses involvement

Various recommendations regarding how to promote HIVST in barbershop businesses focused on monetary incentives for barbershop owners or staff, as they would be the ones conveying the information. The following quotes from participants explain the suggestions to discount barbershop services and to offer financial support for the job of the barbershop business owners:

As a future business owner I would say the only incentive you have, one of the main incentives you should receive, is financial in some form of support for them because it’s a job. It’s a task to spread this kind of message out there so if there is any form of financial support you can provide, I believe that would help them be more motivated for that.

Another participant agreed, and suggested that a percentage of sales from HIVST kits may be used to compensate the barber:

I would agree that the barber who is in charge of presenting the information will get a percentage of the sales that the kits generate.

### Factoring in stigma surrounding HIV and HIVST

Participants explained the benefit of implementing discreet distribution of HIVST kits and inconspicuous packaging of HIVST kits placed in bags with other commonly used contraceptives to increase anonymity and lower shame surrounding HIV. While the barbershop venue was believed to be less stigmatizing given that it is not traditionally associated with HIV testing, participants still emphasized the need for discretion in distribution of HIVST kits:

I feel like there would have to be a lot more extra explaining to them [barbershop clients]. I feel like if you present it in a public setting, they will probably speak to you privately to get the kit. So, I think they would try to be more private about receiving it. You know it takes steps to get people to be really open and comfortable about going to get it in front of all their friends and their peers and stuff.

Another participant agreed and suggested that an HIVST kit may be distributed in conjunction with other sexual health products:

If you put that test in a bag with some magnums (condoms) and some information about preventative care and HIV medicine or whatever you know people will take it… Now as far as buying, ain’t nobody gonna go up and just be like, “Yo let me get that kit,” it’s going to be embarrassing like nobody wants to go in CVS, I guarantee if you put it in the little bag with some magnums… shoot, I would grab it.

### Counseling for post-positive test results and linkage to follow-up care

Several suggestions for dispensing HIVST kits in barbershops emphasized the necessity of providing education to both barbers who distribute the kits and clients who get a positive test result. Participants suggested preparing information on resources for post-positive results, preparing a human presence in the barbershop to answer questions or address concerns, and ensuring that barbers have sufficient knowledge of HIV and HIVST for common conversations with patrons, as demonstrated by the following quotes from two participants:

So, if it was like a pamphlet of information or something that they could also get with general resources that would be good and then you know, it’s not as overwhelming once they start that process. Because they’ll kind of know certain things to look for, just because realistically not everyone has health insurance. So, if they test positive, you know what is this next step going to look like, where do I go from here? If their doctor’s office doesn’t tell them about HMAP (North Carolina HIV Medication Assistance Program) because they don’t know. If that makes sense. Just you know helping them navigate what’s the next step and what’re you going to do. Other than just going to the doctors because if the doctor doesn’t give them good direction or tell them any other good resources available to them then they won’t use them and that’s how you end up losing people or people falling out of care.

One participant echoed the idea that the use of barbershops as an ongoing source of health information was important, and that patrons’ comfortability may increase over time:

But if you have the information set up and as long as the barbers have the resources and are able to talk about it, I feel like that would be truly key. Because I think that what will happen is if you have the booth or something set up people may have questions, but they may not necessarily ask depending on their comfort level with the topic in general, or they may not necessarily have it that particular day. But they can come back the next week when they get a haircut or when they’re in the chair with their barber and kind of have that talk one-on-one you know what was that about and just kind of get a little bit more information, just depending on when that booth gets there and things, they may not be able to ask the questions they’re going to have. But if the Barber can be there with that knowledge, they could be that go-to person.

## Discussion

The purpose of this study was to characterize the acceptability of leveraging barbershop business in Black communities to promote HIV self-testing. Participants’ opinions about HIVST education, promotion, and distribution within barbershops were generally positive, with the majority agreeing that they would self-test if given the chance. Participants recognized the importance of self-testing in diagnosing potential cases at a much faster rate as well as avoiding transmission of HIV to their sexual partners. Barbershop patrons identified the trusting relationship and comfort with their barbers as key factors contributing to the acceptability of receiving HIVST information from them.

These findings are important given the historical and contemporary mistrust among American Black men towards the medical community, stemming from structural racism [38, 53]. The use of nontraditional sources of health information, including peers and trusted community members, has been one strategy used to address Black men’s low uptake of health services [37, 43, 63]. Previous barbershop business-based interventions have yielded positive improvements in health screening and outcomes related to cardiovascular health, vaccinations, physical activity, sexual health, diabetes, and cancer [31, 33, 39, 40, 44, 46].

Participants raised a few concerns that could be addressed through a tailored intervention. Several participants noted the potential cost barrier if participants were expected to pay for the HIVST kit in addition to the services they received barrier if they at the barbershop. This could be easily addressed by connecting interested barbershop clients with the *Together Take Me Home* initiative from the Centers for Disease Control and Prevention, which aims to distribute one million HIVST kits free of charge [64]. An additional concern regarding whether barbers possess sufficient knowledge of HIV to educate their customers could be addressed through comprehensive training for barbers, accompanied by financial incentives, both of which have been utilized in many previous barbershop-based interventions [43]. This training could include information on connecting self-testers to free or low-cost health services following the use of an HIVST for confirmatory testing.

It was also noted by a participant that some barbers may not be comfortable using their shop for health promotion activities. Any future intervention would ensure the full buy-in of barbershop ownership and staff prior to implementation and would incentivize but not mandate participation. Moreover, while barbershops were generally recognized as a non-stigmatizing location for HIV testing and receipt of health information, the possibility for shame and embarrassment still exists in this highly social context. To mitigate this risk, participants suggested bundling HIVST kits with other products for distribution, like condoms. The need to link self-testers to care presents the ongoing challenge of medical mistrust but may be an opportunity for barbershop- based support for facilitated linkage to care. Community-health worker driven interventions may be appropriate for this purpose and could generate collaboration between local departments of health, sexual health clinics, and barbershops.

### Equitable Engagement of Black-Owned Small Businesses by Public Health Practitioners

Participants affirmed the need to consider the challenges of Black-owned small businesses during intervention planning, and stated their belief that engagement of small businesses in an HIVST intervention should be financially remunerative to the business owners and employees. Participants suggested this may come in the form of direct compensation of those helping to deliver the intervention, or in the form of profit sharing if a product, such as an HIVST kit, is sold in a participating barbershop. There are a number of resources on equitable community engagement, including from the DC Center for AIDS Research, that highlight the need for the compensation of individuals, organizations, and businesses who participate in community-based public health initiatives [65–67].

The equitable engagement of Black-owned businesses in public health interventions may benefit from operating under a set of common principles. First, the recognition that Black-owned businesses provide innumerable societal benefits independent of any additional service to their community. Black-owned businesses are more likely to hire Black and minority employees, operate in poor communities, contribute to closing the racial wealth gap, and grow the U.S. economy [68–70]. The survival of these businesses should be acknowledged as critical to addressing racial health disparities, because they are on the frontlines of addressing one of the most central social determinants of health, wealth. Second, given the threats to the long-term sustainability of Black enterprises, participation in health promotion interventions should universally be remunerative for the businesses. This may look different ways depending on the resource constraints of the agencies implementing the interventions. However, creating an ongoing synergistic relationship between Black-owned businesses and public health practitioners is critically important, and innovative ways of equitable community engagement of such businesses should be explored in future research.

## Limitations

There are a few limitations to this study. The results are based on data collected from a small convenience sample of Black men from three separate areas within metropolitan North Carolina. As a result, the findings may not be generalizable to all Black men in the area. Participants were recruited from community-based facilities in North Carolina’s Triangle region (Durham, Raleigh, and Chapel Hill), which may not fully account for regional variations in HIVST acceptability, use, and preferences. Additionally, FGDs were held using Zoom video conferencing, which requires some technological know-how to use. This may have presented a limitation in our recruitment as some participants may have declined to participate based on a lack of availability or knowledge of the technology needed to participate.

## Conclusion

HIVST education and promotion in barbershops is acceptable and feasible among Black men in the Triangle Area of North Carolina. There are high levels of trust between customers and their barber, which presents an opportunity to use barbershops as an alternative source of health education and promotion for a population with high levels of medical mistrust and associated low levels of preventive health service uptake. The use of barbershops as a source of trusted health information is aligned with the cultural position that barbershops have held historically in the Black community. In planning for an HIVST intervention in barbershops, factors like cost, training, and incentivization of implementers are necessary to consider. Furthermore, during the planning and implementation stages of such an intervention, efforts must be made to maximize the benefit of participation to the business. Partnerships between public health actors and the business community must be built on mutual benefit to ensure the long-term viability of these critical initiatives, including HIVST interventions.

## Data Availability

The datasets generated and analyzed during the current study are not publicly available due but are available from the corresponding author on reasonable request.

## Abbreviations

COVID-19: Coronavirus disease 2019
FGD: Focus group discussion
HIV: Human immunodeficiency virus
HIVST: HIV self-testing
PPP: Paycheck Protection Program
